# Cu^II^(atsm) improves the neurological phenotype and survival of SOD1^G93A^ mice and selectively increases enzymatically active SOD1 in the spinal cord

**DOI:** 10.1038/srep42292

**Published:** 2017-02-13

**Authors:** James B. Hilton, Stephen W. Mercer, Nastasia K. H. Lim, Noel G. Faux, Gojko Buncic, Joseph S. Beckman, Blaine R. Roberts, Paul S. Donnelly, Anthony R. White, Peter J. Crouch

**Affiliations:** 1 Department of Pathology, the University of Melbourne, Parkville, Victoria, Australia; 2 Florey Institute of Neuroscience and Mental Health, the University of Melbourne, Parkville, Victoria, Australia; 3 Cooperative Research Centre for Mental Health, Parkville, Victoria, Australia; 4 School of Chemistry, the University of Melbourne, Parkville, Victoria, Australia; 5 Linus Pauling Institute, Department of Biochemistry and Biophysics, Oregon State University, United States; 6 Bio21 Molecular Science and Biotechnology Institute, the University of Melbourne, Parkville, Victoria, Australia

## Abstract

Ubiquitous expression of mutant Cu/Zn-superoxide dismutase (SOD1) selectively affects motor neurons in the central nervous system (CNS), causing the adult-onset degenerative disease amyotrophic lateral sclerosis (ALS). The CNS-specific impact of ubiquitous mutant SOD1 expression is recapitulated in transgenic mouse models of the disease. Here we present outcomes for the metallo-complex Cu^II^(atsm) tested for therapeutic efficacy in mice expressing SOD1^G93A^ on a mixed genetic background. Oral administration of Cu^II^(atsm) delayed the onset of neurological symptoms, improved locomotive capacity and extended overall survival. Although the ALS-like phenotype of SOD1^G93A^ mice is instigated by expression of the mutant SOD1, we show the improved phenotype of the Cu^II^(atsm)-treated animals involves an increase in mature mutant SOD1 protein in the disease-affected spinal cord, where concomitant increases in copper and SOD1 activity are also evident. In contrast to these effects in the spinal cord, treating with Cu^II^(atsm) had no effect in liver on either mutant SOD1 protein levels or its activity, indicating a CNS-selective SOD1 response to the drug. These data provide support for Cu^II^(atsm) as a treatment option for ALS as well as insight to the CNS-selective effects of mutant SOD1.

Amyotrophic lateral sclerosis (ALS) is an adult-onset disease in which motor neurons of the central nervous system (CNS) progressively deteriorate. Initial symptoms are relatively innocuous (e.g. weakness in a hand or slurred speech), but inevitably and relentlessly they escalate. People with ALS become paralysed, lose the ability to breathe, speak and swallow, and due to the absence of an effective treatment most will die within 5 years of diagnosis. The majority of ALS cases are sporadic but approximately 10% are familial and the heritable basis has been ascribed to mutations in over 20 different genes^1^.

Mutations in the copper-dependent antioxidant Cu/Zn-superoxide dismutase (SOD1) were the first described genetic cause of ALS^2^. Since the development of transgenic mice expressing human SOD1 containing ALS-causing substitution mutations^3^,^4^, these mouse models have provided a robust experimental approach to study ALS pathogenesis and progression, as well as opportunity to test new therapeutics in a system that entails basic yet clinically significant features (e.g. a mammalian blood-brain barrier and an adult-onset progressive phenotype). Moreover, mutant SOD1 expressing rodents also recapitulate a salient feature of clinical cases of ALS caused by SOD1 mutations; even though the mutant SOD1 is expressed ubiquitously and persistently from birth, the ALS-like phenotype only presents relatively late in the animals’ life and is the result of selective degeneration of motor neurons in the CNS^3^,^4^. Thus, mutant SOD1-expressing rodents provide opportunity to better understand why a ubiquitously expressed ALS-causing mutation selectively affects the CNS.

In the present study we used transgenic mice expressing human SOD1^G93A^ on a mixed genetic background to assess the therapeutic effects of the metallo-compound Cu^II^(atsm) and to partly investigate how the therapeutic activity of Cu^II^(atsm) may be related to the CNS-selective effects of mutant SOD1 expression. Cu^II^(atsm) – diacetyl*bis(N*(4)-methylthiosemicarbazonato)-Cu^II^ – is a Cu^II^ complex of a *bis*(thiosemicarbazone) ligand^5^ which has been investigated as a potential therapeutic in animal models of ALS and Parkinson’s disease^6^,^7^,^8^,^9^,^10^ and as a PET imaging agent in the clinic for neurological^11^,^12^,^13^ and non-neurological conditions^14^. It is a low molecular weight compound (MW = 321) that is stable (K_A_ = 10^18^) and able to cross the blood-brain barrier^15^. But despite the compound’s stability, an assessment of SOD1^G37R^ mice revealed that approximately 50% of total SOD1 in the spinal cords of these mice exists in a Cu-deficient state, and diminution of this pool following oral administration of Cu^II^(atsm) was shown to involve *in vivo* transfer of Cu from the compound to the Cu-deficient SOD1 in the affected spinal cord^8^. Transfer of Cu from Cu^II^(atsm) to mutant SOD1 was ascribed to at least part of the compound’s therapeutic activity^8^ and this was supported by a subsequent study in which the compound was administered to alternate mutant SOD1 mouse models of ALS^10^.

Thus, biochemical and therapeutic outcomes for Cu^II^(atsm) indicate the compound’s ability to improve Cu bioavailability to SOD1 may contribute, at least in part, to its therapeutic activity in mutant SOD1 mouse models of ALS. Recently, it was demonstrated that the bioavailability of endogenous Cu, but not Zn, is a limiting factor with respect to satiating the elevated requirement for Cu and Zn that is driven by SOD1 over-expression in SOD1^G37R^ mice^16^. Significantly, despite ubiquitous expression of mutant SOD1 in these mice, the insufficient availability of endogenous Cu to SOD1 in these mice is only evident in the CNS^16^. In light of this, and given that the therapeutic activity of Cu^II^(atsm) appears to involve the modulation of Cu bioavailability *in vivo*^8^,^10^, the present study was undertaken to assess whether Cu^II^(atsm) may increase Cu bioavailability to SOD1 in peripheral tissues or only tissues from the CNS. To assess this in the context of the compound’s therapeutic activity, representative CNS (spinal cord) and non-CNS (liver) tissues were collected from SOD1^G93A^ mice in which treating with Cu^II^(atsm) translated into a robust therapeutic effect.

## Results

**Figure 1:**
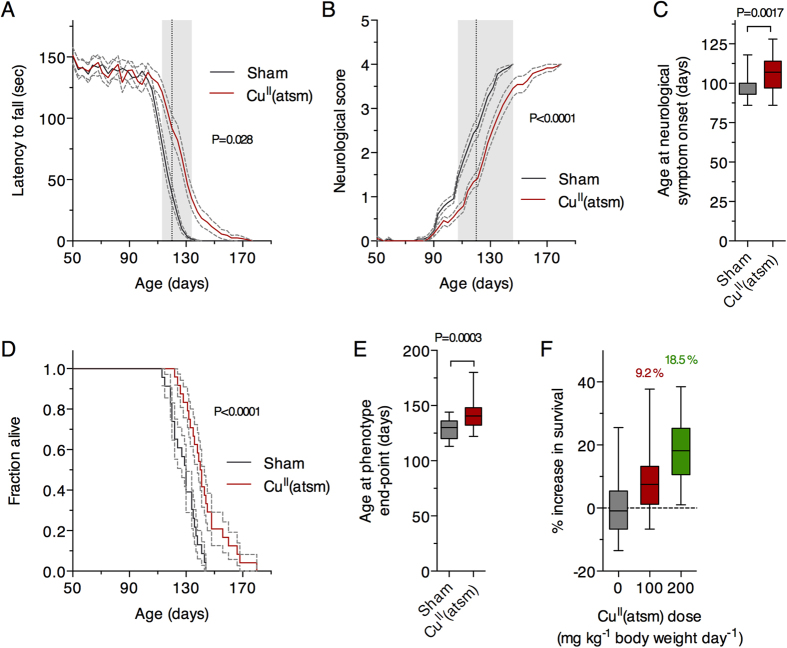
Effect of orally administered Cu^II^(atsm) on neurological phenotype and survival of SOD1^G93A^ mice. (**A**) Performance of sham- and Cu^II^(atsm)-treated SOD1^G93A^ mice on the rotarod test for locomotive function and (**B**) assessment for neurological symptoms via Neurological Score^17^. (**C**) Age of symptom onset defined as an individual mouse attaining a score of 1 in the Neurological Score system^17^. (**D**) Survival to phenotype end-point curves for sham- and Cu^II^(atsm)-treated SOD1^G93A^ mice and (**E**) box and whisker plots showing the overall treatment effect on survival. (**F**) Relationship between total daily dose of Cu^II^(atsm) and percentage increase in mean survival. Data for the 100 mg dose are calculated from experiment presented in (**D**) (mean = 143 days, n = 24), data for the 200 mg dose calculated from experiments in Williams *et al*.^10^ (mean = 155 days, n = 20), and data for the 0 mg dose calculated across the two studies (mean = 131 days, n = 44). Solid lines in (**A** and **B**) are mean values. Grey dashed lines in (**A**,**B** and **D**) represent SEM. Data in (**C**,**E** and **F**) are presented as box (median ± 95% CI) and whisker (maximum and minimum) plots. P values in (**A** and **B**) represent statistical significance of the treatment effect (repeat measures ANOVA), whereas grey shaded boxes indicate periods for statistically significant differences between mean values for sham- and Cu^II^(atsm)-treated mice (Sidak’s multiple comparisons test). P values in (**C** and **E**) indicate a statistically significant difference between mean values for sham- and Cu^II^(atsm)-treated mice (unpaired t-test). P value in (**D**) represents statistically significant treatment effect (Cox proportional hazards model). Percentage values in F represent mean increase in survival for each Cu^II^(atsm) dose. For A-E, n = 23 sham-treated mice and n = 24 Cu^II^(atsm)-treated mice (treatments administered twice daily by gavage with Cu^II^(atsm) administered per dose at 50 mg kg^−1^ mouse body weight). Vertical dashed lines in A and B represent the age at which a separate cohort of mice was killed for biochemical analyses.

Litter- and gender-matched SOD1^G93A^ mice on a mixed genetic background were treated daily with Cu^II^(atsm) or sham control from the age of 50 days. Twice weekly assessment on the rotarod test revealed a sharp decline in locomotive function commencing when the mice were around 100 days old (Fig. 1A). This decline was delayed in mice that were treated with Cu^II^(atsm), with the treatment effect attaining statistical significance at 113 days then persisting for the remainder of the study period. An alternate assessment of neurological function^17^ provided a comparable outcome; the neurological phenotype of the SOD1^G93A^ mice noticeably and progressively worsened from around 100 days but treating with Cu^II^(atsm) delayed the phenotype (Fig. 1B). The latter of these two methods for assessing phenotype progression revealed that the Cu^II^(atsm) treatment delayed neurological symptom onset under the present experimental conditions by 9 days (Fig. 1C).

The improved neurological phenotype of SOD1^G93A^ mice in response to the Cu^II^(atsm) treatment translated to an improvement in overall survival to phenotypic end-point (Fig. 1D). Treating with Cu^II^(atsm) increased median survival by 8% from 130 to 141 days and mean survival by 11% from 129 to 143 days (Fig. 1E). The comparable effect that Cu^II^(atsm) had on delaying neurological onset and extending survival to phenotypic end-point equated to no change in the duration of symptom progression: on average, the period of symptom progression from onset to end-point was 16 days in the sham-treated mice and 15 days in the Cu^II^(atsm)-treated mice (P = 0.85, two-tailed t-test). These results are consistent with a previous study in which ALS mice expressing SOD1 with the G37R mutation^4^ were treated orally with Cu^II^(atsm)^8^ and a more recent study in which Cu^II^(atsm) was administered to SOD1^G93A^ mice via a transdermal route^10^. Significantly, despite fundamental differences in the route of administration and performing the experiments across two different colonies of mice at two different institutes, doubling the daily dose effectively doubled the extension in survival elicited by administering Cu^II^(atsm) to SOD1^G93A^ mice (Fig. 1F).

**Figure 2:**
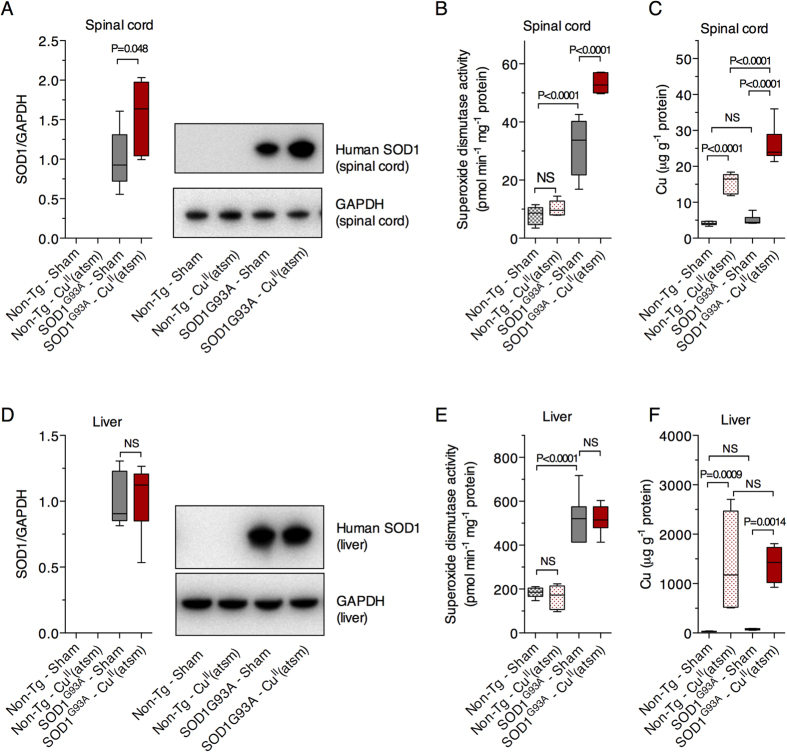
The effect of orally administered Cu^II^(atsm) on mutant SOD1 and Cu levels in spinal cords and livers of SOD1^G93A^ mice. Relative abundance of mutant SOD1 protein in spinal cord (**A**) and liver (**D**) samples determined via western blot using an antibody that detects only human SOD1. Mutant SOD1 protein levels are expressed relative to the loading control GAPDH. Representative western blot images are shown. SOD1 activity in TBS-soluble extracts from mouse spinal cords (**B**) and livers (**E**) presented as pmol superoxide decay min^−1^ mg^−1^ tissue protein. The amount of Cu g^−1^ protein in spinal cord (**C**) and liver (**F**) tissue. Treatments were administered twice daily by gavage and commenced when the mice were 50 days old. Cu^II^(atsm) administered per dose was 50 mg kg^−1^ mouse body weight. Mice were killed at 120 days old to collect tissues for analysis. Graphed data are box (median ± 95% CI) and whisker (maximum and minimum) plots and P value represents statistically significant treatment effect on mean values (unpaired t-test in (**A** and **D**) or one-way ANOVA with Tukey’s multiple comparisons test in (**B**,**C**,**E** and **F**)). NS = not statistically different. For all data shown, n = 6 mice per treatment group.

Assessing the influence of Cu^II^(atsm) on levels of mutant SOD1 protein in spinal cord tissue from SOD1^G93A^ mice at the mid-stages of symptom progression (indicated via vertical dashed lines in Fig. 1A and B) demonstrated that treating with Cu^II^(atsm) increased levels of mutant SOD1 in the disease-affected CNS tissue (Fig. 2A). Catalytic activity of SOD1 is dependent upon the protein binding Cu^18^. Thus, we measured SOD1 activity in spinal cord extracts from sham- and Cu^II^(atsm)-treated mice to assess whether the increase in mutant SOD1 protein in response to the Cu^II^(atsm) translated to an increase in SOD1 activity. Reflecting overall differences in SOD1 protein levels between non-transgenic mice and the over-expressing SOD1^G93A^ mice^19^, SOD1 activity was relatively low in extracts collected from non-transgenic mice and this was unchanged by the Cu^II^(atsm) treatment (Fig. 2B). As a result of human SOD1 overexpression, and because the G93A mutation does not affect the enzyme’s dismutase activity^3^,^20^, SOD1 activity was relatively high in the spinal cords of the sham-treated SOD1^G93A^ mice (Fig. 2B). This was further increased by the Cu^II^(atsm) treatment (Fig. 2B). Moreover, analysing the Cu content of spinal cords supported outcomes from the SOD1^G37R^ model^8^; elevated spinal cord Cu in Cu^II^(atsm)-treated non-transgenic mice confirmed that oral administration of the compound affects Cu levels in the CNS, and the same dose administered to mice expressing mutant SOD1 elicits a greater response (Fig. 2C). In contrast to these effects in the spinal cord, administering Cu^II^(atsm) to SOD1^G93A^ mice had no influence on mutant SOD1 protein levels or activity in the liver (Fig. 2D,E), nor was there any statistically significant difference between non-transgenic and SOD1^G93A^ mice with respect to liver Cu levels in response to the Cu^II^(atsm) treatment (Fig. 2F, P = 0.99).

**Figure 3:**
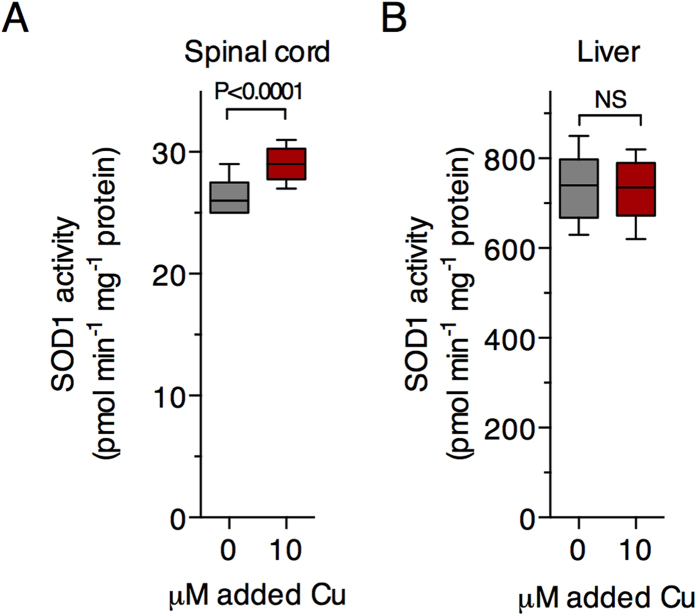
The effects of adding Cu^2+^ directly to tissue extracts from SOD1^G93A^ mice on SOD1 activity. SOD1 activity in TBS-soluble extracts from SOD1^G93A^ mouse spinal cords (**A**) and livers (**B**) presented as pmol superoxide decay min^−1^ mg^−1^ tissue protein. Tissue extracts were prepared from untreated SOD1^G93A^ mice killed at 120 days old. All data are presented as box (median ± 95% CI) and whisker (maximum and minimum) plots and P values represent statistically significant differences between mean values for indicated groups (paired t-test). NS = not statistically different. For all data shown, n = 6 mice per treatment group.

The increase in SOD1 activity in the spinal cords of Cu^II^(atsm)-treated SOD1^G93A^ mice (Fig. 2B) is supportive of reports which confirm the presence of a large pool of Cu-deficient SOD1 in the spinal cords of SOD1^G93A^ and SOD1^G37R^ mice^8^,^10^ and that *in vivo* transfer of Cu from Cu^II^(atsm) to SOD1 can increase the concentration of Cu-containing SOD1, ergo its Cu-dependent dismutase activity^8^,^10^. The absence of any change to SOD1 activity in the livers of Cu^II^(atsm)-treated SOD1^G93A^ mice (Fig. 2E) by contrast, indicates that endogenous Cu bioavailability in the liver is able to meet the elevated requirement for Cu due to SOD1 over-expression and that SOD1 in the livers of the transgenic mice is therefore relatively Cu-replete (a possibility supported recently^16^), or that Cu delivered as Cu^II^(atsm) does not become bioavailable to SOD1 in the liver. To partly interrogate these possibilities, we adopted a protocol in which Cu^2+^ ions are added to tissue extracts in order to assess whether SOD1 activity in the extracts is responsive to the available Cu^21^. Outcomes from this assay showed SOD1 activity in SOD1^G93A^ mouse spinal cord extracts is increased by directly adding Cu^2+^ ions to the tissue extract (Fig. 3A) but activity in liver extracts from the same mice is not (Fig. 3B).

**Figure 4:**
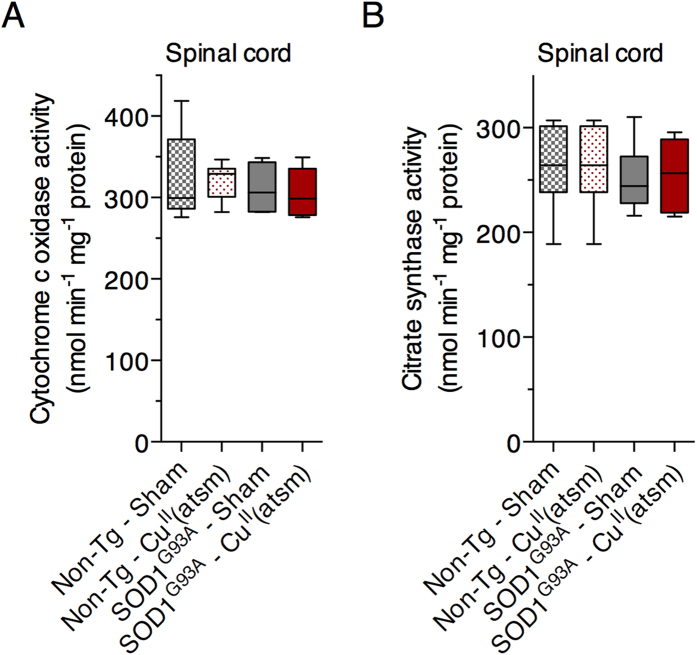
Effect of orally administered Cu^II^(atsm) on mitochondrial cytochrome c oxidase and citrate synthase activity in SOD1^G93A^ mice. (**A**) Cytochrome c oxidase activity in non-transgenic and SOD1^G93A^ mouse spinal cords presented as nmol cytochrome c oxidised min^−1^ mg^−1^ tissue protein. (**B**) Citrate synthase activity in non-transgenic and SOD1^G93A^ mouse spinal cords presented as nmol DTNB reduced min^−1^ mg^−1^ tissue protein. Treatments were administered twice daily by gavage and commenced when the mice were 50 days old. Cu^II^(atsm) administered per dose was 50 mg kg^−1^ mouse body weight. Mice were killed at 120 days old to collect tissues for analysis. Graphed data are box (median ± 95% CI) and whisker (maximum and minimum) plots. No statistically significant differences exist between any of the treatment groups (one-way ANOVA with Tukey’s multiple comparisons test). For all data shown, n = 6 mice per treatment group.

Other important enzymes are dependent upon Cu for their catalytic activity, including cytochrome c oxidase, the terminal enzyme complex of the mitochondrial electron transfer chain. Consistent with a recent report^10^, cytochrome c oxidase activity is unaltered in the spinal cords of SOD1^G93A^ mice and treating with Cu^II^(atsm) has no detectable influence on its activity in these mice (Fig. 4A).

**Figure 5:**
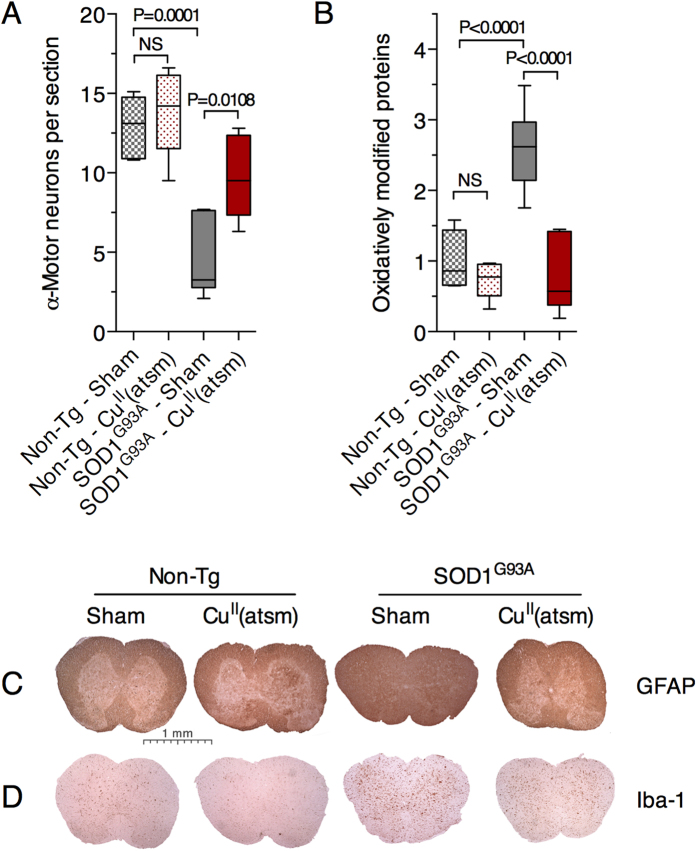
Effect of orally administered Cu^II^(atsm) on α-motor neurons, oxidative damage and astrogliosis in spinal cords of SOD1^G93A^ mice. (**A**) Quantitation of α-motor neurons per section in both ventral horn regions of spinal cord sections determined via cresyl violet staining. Only motor neurons with a diameter equivalent to 20 μm or greater were counted. (**B**) Abundance of oxidatively modified proteins determined using the OxyBlot assay in spinal cord tissue expressed relative to levels detected in sham-treated non-transgenic controls. Representative histology images for GFAP (**C**) and Iba-1 (**D**) immunoreactivity in spinal cord transverse sections. Data in (**A** and **B**) are presented as box (median ± 95% CI) and whisker (maximum and minimum) plots. P values represent statistically significant differences between mean values for indicated groups (one-way ANOVA with Tukey’s multiple comparisons test, n = 6 mice per treatment group). NS = not statistically different.

A multitude of dysfunctional pathways appear to contribute to symptom onset and progression in ALS. Considering that SOD1 activity is already higher in the spinal cords of the sham-treated mutant SOD1 mice due to over-expression of the transgene (Fig. 2B)^3^,^4^,^8^,^16^, and notwithstanding the presence of large pools of Cu-deficient and catalytically inactive SOD1, it is therefore unlikely that increasing SOD1 activity in the spinal cords of mutant SOD1 over-expressing mice *per se* is solely responsible for the Cu^II^(atsm) induced improvement in the animals’ phenotype. Supporting this, our assessment of broad indications of spinal cord tissue health (oxidative damage, astrogliosis and motor neuron numbers) all demonstrated the beneficial effects of Cu^II^(atsm) in the primary site of pathology in the SOD1^G93A^ mice (Fig. 5).

## Discussion

Mutant SOD1 is a cause of familial ALS^2^ and transgenic mice expressing the mutant protein accurately recapitulate many features of the disease^3^,^4^. Significantly, this includes the onset of symptoms of motor neuron decline in adulthood, even though the causative mutation is expressed ubiquitously and persistently from birth. But to date, an unequivocal explanation for why ubiquitously expressed mutant SOD1 selectively affects the CNS in mice and humans has remained elusive.

In the present study, and in the context of the therapeutic agent Cu^II^(atsm), we investigated the bioavailability of Cu as a potential contributing factor. The over-expression of mutant SOD1 in transgenic mice disrupts Cu homeostasis; some studies indicate increased levels of spinal cord Cu in multiple mutant SOD1 mouse models of ALS^22^ and the abundance of various Cu transporters and Cu chaperones is also altered^22^,^23^. Furthermore, the potential to improve the symptoms of ALS and protect motor neurons in the CNS via therapeutic strategies that modulate Cu bioavailability has already been demonstrated^24^; treating mutant SOD1 mice with Cu-chelating agents such as ammonium tetrathiomolybdate and D-penicillamine or with the Cu-delivery agent Cu^II^(atsm) improves their neurological phenotype and extends survival^7^,^8^,^9^,^10^,^22^,^25^,^26^,^27^,^28^. Collectively these outcomes lend support to the notion that Cu bioavailability is an important factor in the ALS-like symptoms that develop in mutant SOD1 mice. Details of the deleterious mechanistic processes are yet to be elucidated, but the emerging consensus appears to be that disrupted Cu bioavailability, rather than Cu deficiency or Cu accumulation *per se*, is a primary feature of the neurodegenerative process.

This is consistent with some aspects of the potential SOD1 gain of function in ALS. SOD1 is a well-characterised metalloenzyme with a relative abundance of biochemical and biophysical information on its interaction with Cu and Zn. These interactions govern the protein’s maturation, stability and structure^18^,^29^,^30^,^31^,^32^, and Cu-associated perturbations to SOD1 maturation can promote aggregation via their differential effects on the seeding and growth of SOD1 fibrils^33^. This implicates Cu in the widely supported notion that SOD1 mis-folding and aggregation is a primary mechanism of toxicity for SOD1 in mutant SOD1 cases of ALS^34^,^35^. Further to this, altered interaction with Cu also provides a plausible mechanism by which SOD1 may contribute to motor neuron decline in sporadic cases of ALS that do not involve mutant SOD1; even in the absence of a disease-causing mutation, the bioavailability of Cu to SOD1 is an important determinant of the protein’s stability and structure^36^, and mis-folded and aggregated SOD1 is present in sporadic cases of ALS^37^. Moreover, the presence of Cu-deficient SOD1 in the disease-affected spinal cords of ALS model mice has been confirmed; direct assessment of metals bound to SOD1 via a quantitative mass spectrometry approach^38^ shows that almost half of the total SOD1 pool in the spinal cords of SOD1^G37R^ mice is Cu-deficient and a similar pool of Cu-deficient SOD1 is present in the spinal cords of SOD1^G93A^ mice^10^.

But although the role for disrupted Cu bioavailability in the pathogenesis of ALS is supported by several lines of evidence, a Cu-centric explanation for why the CNS is more susceptible to the effects of mutant SOD1 expression is less clear. In the present study we show that oral treatment with Cu^II^(atsm) improves the neurological phenotype and survival of SOD1^G93A^ mice (Fig. 1) and that the treatment increases the abundance of mutant SOD1 in the spinal cord (Fig. 2). These results are consistent with outcomes from previous studies^10^ and the increase in SOD1 activity in the spinal cord (Fig. 5A) is consistent with the demonstrated capacity for Cu^II^(atsm) to make Cu bioavailable to SOD1 *in vivo*^8^. Overall, the *in vivo* effects for Cu^II^(atsm) are consistent across multiple mutant SOD1 murine models of ALS and to date is reproduced via two distinct drug administration methods (summarised in Table 1). Across multiple studies it therefore appears that Cu^II^(atsm) stabilises mutant SOD1 *in vivo*, in a seemingly non-toxic form, by satiating its requirement for Cu and converting Cu-deficient SOD1 to mature holo-SOD1.

But in contrast to these observations in the spinal cord, treating with Cu^II^(atsm) had no influence on SOD1 levels or activity in the liver (Fig. 2D–F), indicating that SOD1 in the livers of SOD1^G93A^ mice is relatively Cu-replete and/or that Cu^II^(atsm) does not make Cu bioavailable to SOD1 in the liver. Our observation that supplementing tissue extracts with Cu increased SOD1 activity in the spinal cord but not the liver (Fig. 3) lends support to the former of these possibilities as does our recent assessment of SOD1 in SOD1^G37R^ mice^16^. Due to ubiquitous expression of the transgene, SOD1 protein levels are elevated in various CNS and non-CNS tissues from SOD1^G37R^ mice, and in the non-CNS tissues this increase in SOD1 protein is matched by a commensurate increase in SOD1 activity as well as a commensurate increase in Cu and Zn^16^. However, in the CNS tissues, although the increased level of SOD1 protein is matched by an increase in Zn, a comparable increase in Cu is not evident. As a result, the Cu-dependent activity of SOD1 in the CNS tissue is limited^16^.

It therefore appears that while the increased requirement for Cu due to SOD1 over-expression is met in non-CNS tissues, the natural bioavailability of Cu in CNS tissues is a limiting factor, leading to an accumulation of Cu-deficient SOD1 only in CNS tissue. This may, in part, be related to the endogenous pathways via which Cu is presented to SOD1. A key stage in SOD1 maturation involves the Cu chaperone for SOD1 (CCS) which acquires Cu for delivery to SOD1 and thereby facilitates SOD1 disulphide bond formation for structural stability. Endogenous mouse CCS appears relatively inefficient at facilitating human SOD1 maturation^10^. As such, under conditions whereby the natural bioavailability of CNS Cu becomes a rate limiting factor in human SOD1 over-expressing mice, the relative inefficiency of endogenous mouse CCS could become an additive exacerbating factor. Indeed, alleviating the limited availability of Cu to SOD1 via treating with Cu^II^(atsm) induces a relatively modest increase in the Cu content of SOD1 in the spinal cords of SOD1^G93A^ mice (and a relatively modest increase in mouse survival), but when the same treatment is applied to SOD1^G93A^ mice that also express human CCS the effect on Cu delivery to mutant SOD1 (and on mouse survival) is dramatic^10^. Thus, when human CCS is expressed in SOD1^G93A^ mice the inefficiency of Cu delivery to human SOD1 in the spinal cord is no longer an impediment, and increasing spinal cord Cu via Cu^II^(atsm) therefore improves survival of the mutant SOD1 expressing mice to a remarkable extent.

Consistent with outcomes from previous studies which utilised alternate mutant SOD1 mouse models of ALS^8^,^9^, we show here that treating with Cu^II^(atsm) potently decreases protein markers of oxidative stress in the SOD1^G93A^ mice (Fig. 5B) and that markers of astrogliosis are also diminished (Fig. 5C,D). While the explicit source of oxidative stress leading to oxidative damage in the SOD1^G93A^ mice is yet to be unequivocally demonstrated, disruptions to physiological electron flux through the mitochondrial respiratory chain is a widely mooted possibility^39^,^40^. Cytochrome c oxidase, complex IV of the respiratory chain, requires Cu for its catalytic activity. As such, and in the context of modulating Cu bioavailability as potential part of the mechanism of action for Cu^II^(atsm) in the mutant SOD1 mice, this raises the possibility that an unmet requirement for Cu in cytochrome c oxidase could contribute to respiratory chain dysfunction, ergo oxidative stress, in the SOD1^G93A^ mice. However, our analysis of cytochrome c oxidase activity in the spinal cords of these mice indicates no overt impediment to this Cu-dependent aspect of mitochondrial function (Fig. 4) and this is consistent with outcomes reported in a recent study for mice that only express mutant SOD1^10^. However, decreased cytochrome c oxidase activity has been reported in mice expressing mutant SOD1. Co-expression of human Cu chaperone for SOD1 (CCS) with mutant SOD1 dramatically accelerates the ALS-like phenotype of the mutant SOD1 mice and induces a mitochondrial pathology^10^,^41^. Cytochrome c oxidase activity is decreased by 75% in the CCS x SOD1^G93A^ mice yet is completely restored by treating with Cu^II^(atsm)^10^. Thus, while treating with Cu^II^(atsm) restores functionality to cytochrome c oxidase in SOD1^G93A^ x CCS mice^10^, outcomes from the present study do not implicate Cu-dependent cytochrome c oxidase activity in the observed changes in oxidative stress in the Cu^II^(atsm)-treated SOD1^G93A^ mice.

The presence of a substantial pool of Cu-deficient SOD1 in the spinal cord but not liver could explain the apparent tissue-specific effect that Cu^II^(atsm) has on overall levels of Cu in each tissue and this, in turn, could have implications for the clinical use of Cu^II^(atsm) as a PET imaging agent. Treating with Cu^II^(atsm) resulted in a greater elevation in Cu levels in the spinal cord of SOD1^G93A^ mice when compared to non-transgenic mice yet there was no significant difference between SOD1^G93A^ and non-transgenic mice with respect to the liver (Fig. 2C and F). Cu^II^(atsm) labelled with positron emitting Cu isotopes shows greater retention of the signal in the motor cortex of ALS patients^13^ as well as disease-specific regions of the Parkinson’s disease-affected^12^ brain and the brains of people affected by MELAS (mitochondrial myopathy, encephalopathy, lactic acidosis and stroke-like episodes)^11^. The biochemical mechanisms that may govern selective retention of the tracer in the disease-affected regions have been investigated and include oxidative stress, hypoxia and mitochondrial respiratory chain dysfunction^42^,^43^. Central to these mechanisms is the presence of cellular proteins which will bind, and therefore retain, the Cu after is has dissociated from the atsmH_2_ scaffold^44^. Many proteins under physiological conditions will be able to bind Cu should cellular Cu levels rise relatively rapidly (e.g. metallothioneins), but it stands to reason that cells containing a higher concentration of Cu-deficient proteins will have a greater capacity to retain Cu under such conditions. A substantial pool of Cu-deficient SOD1 exists in CNS tissue from mutant SOD1 expressing mice^8^,^10^, and data presented here (Fig. 3) and previously^16^ indicate the accumulation of Cu-deficient SOD1 in these animals is most evident in CNS tissue.

## Methods

### Cu^II^(atsm) treatment of SOD1^G93A^ mice

All research involving live mice was approved by a University of Melbourne Animal Experimentation Ethics Committee (#1312908) and conformed with guidelines of the Australian National Health and Medical Research Council. Hemizygous mice expressing a transgene for human SOD1 containing the G93A substitution mutation (SOD1^G93A^) on the mixed B6/SJL background were from the Jackson Laboratories (strain B6SJL-Tg(SOD1*G93A)1GurJ) and generously provided by Prize4Life. Non-transgenic littermates were used as a control. Prior to treating, mice were allocated based on sex and litter to either the “survival” cohort or the “biochemical” cohort. Mice in the survival cohort were kept through to phenotypic end-point to collect data on the effects of treatment on survival and symptom progression, and mice in the biochemical cohort were killed at the age of 120 days to collect tissues for biochemical analyses. Within each cohort individual mice were allocated based on sex and litter to either the sham treatment or the Cu^II^(atsm) treatment group. All treatments were thus spread evenly across sexes, litters and genotypes. Treatment commenced when the mice were 50 days old. Sham treatment involved gavage with standard suspension vehicle (SSV; 0.9% w/v NaCl, 0.5% w/v Na-carboxymethylcellulose, 0.5% v/v benzyl alcohol, 0.4% v/v Tween-80). Cu^II^(atsm) treatment involved gavage using SSV supplemented with Cu^II^(atsm). Cu^II^(atsm) was synthesised as described previously^5^,^45^. Dose of Cu^II^(atsm) administered to each animal was 50 mg kg^−1^ body weight. Treatments were administered twice daily, 7 days week^−1^ through to phenotypic end-point (survival cohort) or until the mice reached 120 days of age (biochemical cohort).

### Phenotype assessment of mice

SOD1^G93A^ mice were assessed for symptom progression using the rotarod assay for locomotive function and a Neurological Score system previously described^17^. Mice were habituated to the rotarod assay for 5 days prior to recording performance. During the recording period the rotation speed of the rotarod was accelerated from 4 rpm to 40 rpm over a 180 second period with the time taken to fail the task (latency to fall) recorded for each mouse. During assessment each mouse was subjected to two independent runs on the rotarod and only the higher latency to fall score used for subsequent analysis. Survival of SOD1^G93A^ mice represents the age at which an individual mouse could no longer right itself within 15 seconds of being placed on its side. All phenotype assessments were performed by researchers blinded to mouse genotype and treatment.

### Mouse tissue collection

SOD1^G93A^ mice and non-transgenic littermates at 120 days old were anaesthetised by intraperitoneal injection of ketamine (120 mg kg^−1^) and xylazine (16 mg kg^−1^) in PBS, then perfused transcardially with PBS containing 0.25% (v/v) phosphatase inhibitor cocktail 2 (Sigma), 1% (v/v) Complete EDTA-free protease inhibitor (Roche), and 20 U mL^−1^ heparin (Sigma). Following perfusion, tissues were excised then (excepting regions of spinal cord used for histology as described below) snap frozen on dry ice and stored at −80 °C.

### SDS-PAGE and western blotting

Tissue samples were homogenised using polypropylene pestles in TBS supplemented with 0.5% (v/v) phosphatase inhibitor cocktail 2 (Sigma), 2% (v/v) Complete EDTA-free protease inhibitor (Roche), and 5% (v/v) DNAse. Homogenates were then separated into TBS-soluble and TBS-insoluble fractions by centrifugation (18,000× g, for 30 minutes at 4 °C). TBS-soluble extracts were prepared in denaturing sample buffer containing 62.2 mM Tris, 5% (v/v) glycerol, 2% (w/v) SDS, and 0.0025% (w/v) bromophenol blue prior to loading onto 4–12% NuPAGE Novex Bis-Tris Midi gels (Life Technologies) and electrophoresis at 200 V for 40 minutes in MES SDS running buffer (Life Technologies). Resolved proteins were transferred onto PVDF membranes using iBlot gel transfer stacks (Life Technologies) as per manufacturer’s instructions. Membranes were blocked for 1 hour in PBS supplemented with 0.05% (v/v) Tween-20 (Chemsupply) and 4% (w/v) skim milk powder prior to incubation with primary antibodies in blocking buffer, overnight at 4 °C. Primary antibodies used were raised to detect human SOD1 (Abcam; 1:100,000) or GAPDH (Cell Signaling; 1:5,000). A horseradish peroxidase-conjugated secondary antibody for anti-rabbit IgG (Cell Signaling; 1:5,000) was then used, and subsequent immunoreactive protein bands visualised by adding Enhanced Chemiluminescence (ECL Advance, GE Healthcare) to membranes and detecting luminescence using a FujiFilm LAS-3000 imager. Quantitation of immunoreactivity was performed using ImageJ software on TIFF file images.

### SOD1 activity

SOD1 activity in TBS-soluble tissue extracts (described above) was assessed via a pyrogallol assay based on published procedures^46^,^47^. Pyrogallol (Sigma) was added to a reaction buffer (50 mM Tris, 1 mM EGTA, pH 7.4) to a final concentration of 200 μM and allowed to equilibrate for 1 minute. TBS-soluble tissue extracts were added then reaction mixtures monitored at 325 nm. Reaction mixtures supplemented with 10 mM KCN were used to determine the KCN-sensitive activity attributable to SOD1. SOD1 activity was determined by calculating the rate of change through the linear phase of reaction. Reactions mixtures containing equivalent volumes of TBS homogenising buffer ±KCN were included as additional controls. For experiments in which tissue extracts were supplemented with Cu^2+^ (Fig. 3), TBS-soluble extracts were incubated with CuCl_2_ (final Cu^2+^ concentration = 10 μM) overnight at 4 °C before performing the activity assay.

### Tissue copper analysis

Sections of frozen tissue were weighed then analysed for total copper levels following protocols described previously^42^. Briefly, tissue samples were homogenised in TBS as described above then aliquots assessed for total protein content. The remainder of the homogenate was dried down, digested using concentrated nitric acid, then analysed for copper content using an Agilent 7700 Series ICP-MS with a helium reaction cell.

### Cytochrome c oxidase and citrate synthase activity

TBS-insoluble spinal cord material was solubilised by adding lauryl maltoside to a final concentration of 1.5% (v/v). Lauryl maltoside soluble extracts were recovered by centrifugation (21,000× g, 3 minutes, 4 °C) then normalised to a consistent protein concentration. Cytochrome c oxidase and citrate synthase activities were determined as described previously^48^.

### Histology

All histology protocols were as previously described^8^. Briefly, lumbar regions of mouse spinal cord freshly dissected from mice at 120 days old were submersion fixed in 4% (v/v) paraformaldehyde, paraffin-embedded, then sectioned and mounted onto glass microscope slides. Sections were stained with cresyl violet for motor neuron counts or incubated with primary antibodies to GFAP (Dako) or Iba-1 (Wako) for assessing astrogliosis. Motor neuron values presented per mouse represent the average number of α-motor neurons from approximately 30 separate sections per mouse (spanning approximately 2 mm along the longitudinal plane of the spinal cord). For all motor neurons counted, the area was quantified using Image J software and only those motor neurons with an area equivalent to a 20 μm diameter or greater were considered as α-motor neurons. Data presented in Fig. 5A represent the average number of α-motor neurons in both ventral horn regions of the grey matter per section.

### Oxidatively modified proteins

TBS-soluble spinal cord samples were analysed for oxidatively modified proteins using the OxyBlot Protein Oxidation Detection kit (Millipore) as described previously^8^.

### Statistical analyses

Data sets were assessed for statistical significance of the Cu^II^(atsm) treatment on age-related outcomes via the following tests: two-tailed repeat measures ANOVA (Fig. 1A,B); Cox proportional hazards model (Fig. 1D). Gender and litter were both excluded as potential confounding factors in the proportional hazards model. Data sets were assessed for the statistical significance of the Cu^II^(atsm) treatment on static group means via the following tests: two-tailed t-test (Figs 1C,E and 2A,D); ordinary one-way ANOVA with Tukey’s multiple comparisons test (Figs 2B,C,E,F, 4A,B and 5A,B); two-tailed paired t-test (Fig. 3A,B). Experimental replicates are individual mice or tissues collected from individual mice. Phenotype assessment data shown in Fig. 1 involved n = 23 sham-treated mice (12 female, 11 male) and n = 24 Cu^II^(atsm)-treated mice (13 female, 11 male). Biochemical data shown in Figs 2, 3, 4 and 5 involved tissues from n = 6 mice (3 female, 3 male) for each treatment group.

## Additional Information

**How to cite this article**: Hilton, J. B. *et al*. Cu^II^(atsm) improves the neurological phenotype and survival of SOD1^G93A^ mice and selectively increases enzymatically active SOD1 in the spinal cord. *Sci. Rep.* **7**, 42292; doi: 10.1038/srep42292 (2017).

**Publisher's note:** Springer Nature remains neutral with regard to jurisdictional claims in published maps and institutional affiliations.

## Figures and Tables

**Table 1:** Summary of therapeutic outcomes for Cu^II^(atsm) across multiple mutant SOD1 mouse models of ALS.

Study	Mouse model	Genetic background	Age when treatment commenced	Daily dose (mg kg^−1^ body weight)	Administration route	Increase in survival#
Soon *et al*.^9^	Low copy SOD1^G93A^	Congenic; C57BL6	140 days	30 (5 days week^−1^)	Oral	14%
Soon *et al*.^9^	Low copy SOD1^G93A^	Congenic; C57BL6	200 days	30 (5 days week^−1^)	Oral	10%
McAllum *et al*.^7^	High copy SOD1^G37R^	Congenic; C57BL6	40 days	10 (7 days week^−1^)	Oral	8%
McAllum *et al*.^7^	High copy SOD1^G37R^	Congenic; C57BL6	40 days	30 (7 days week^−1^)	Oral	18%
McAllum *et al*.^7^	High copy SOD1^G37R^	Congenic; C57BL6	40 days	60 (7 days week^−1^)	Oral	26%
McAllum *et al*.^7^	High copy SOD1^G37R^	Congenic; C57BL6	149 days*	60 (7 days week^−1^)	Oral	12%
Roberts *et al*.^8^	High copy SOD1^G37R^	Congenic; C57BL6	40 days	30 (7 days week^−1^)	Oral	18%§
Williams *et al*.^10^	High copy SOD1^G93A^	Mixed; B6SJL	5 days	200 (7 days week^−1^)	Transdermal	25%
Williams *et al*.^10^	High copy SOD1^G93A^	Mixed; B6SJL	50 days	200 (7 days week^−1^)	Transdermal	19%
Williams *et al*.^10^	High copy SOD1^G93A^ x CCS	Mixed; B6SJL	Prenatal	60 (7 days week^−1^)	Transdermal	2,800%
Present study	High copy SOD1^G93A^	Mixed; B6SJL	50 days	100 (7 days week^−1^)	Oral	9%

^#^Increase in mean survival relative to sham treated mice.

^*^Treatment commenced after individual mice lost 20% of their basal pre-symptom functionality on the rotarod test for locomotive function and 149 days is the mean age at which all mice included in the study reached this phenotypic criterion.

^§^Performed concurrently with the McAllum *et al*. study. These survival data were presented in the McAllum *et al*. paper and therefore not re-published in the Roberts *et al*. paper which instead reported other therapeutic outcomes and mechanism of action data.
